# Emerging Biomarkers in Pediatric Food Allergy: From Mechanistic Endotyping to Precision Diagnosis and Therapeutic Monitoring

**DOI:** 10.3390/biomedicines14071608

**Published:** 2026-07-17

**Authors:** Enrico Vito Buono, Nicolò Canducci, Roberta Carbone, Marialaura Menzella, Anna Montanari, Tommaso Carretta, Valentina Fainardi, Carlo Caffarelli, Susanna Esposito

**Affiliations:** Pediatric Clinic, University Hospital of Parma, 43126 Parma, Italy; enricovito.buono@unipr.it (E.V.B.); nico_canducci@hotmail.it (N.C.); roberta.carbone2@unipr.it (R.C.); marialaura.menzella21@gmail.com (M.M.); anna.montanari@unipr.it (A.M.); tommaso.carretta@unipr.it (T.C.); valentina.fainardi@unipr.it (V.F.); carlo.caffarelli@unipr.it (C.C.)

**Keywords:** pediatric food allergy, biomarkers, basophil activation test, epithelial barrier dysfunction, gut microbiota, precision medicine

## Abstract

**Background:** Food allergy is a heterogeneous pediatric disease involving IgE-mediated, non-IgE-mediated, and mixed immune mechanisms, with manifestations ranging from mild symptoms to life-threatening anaphylaxis. Current diagnostic tools, including clinical history, skin prick testing, serum-specific IgE measurement, and oral food challenge, have limitations in specificity, invasiveness, prognostic value, and ability to guide personalized management. **Methods:** This narrative review summarizes emerging biomarkers in pediatric food allergy and evaluates their diagnostic, prognostic, predictive, and therapeutic potential. A literature search was conducted in PubMed/MEDLINE and Cochrane Central for English-language studies published between December 2015 and March 2026. Eligible studies included original clinical or translational research involving children aged 0–18 years and assessing functional cellular assays, epithelial barrier markers, intestinal permeability, gut microbiota, metabolomics, transcriptomics, proteomics, epigenetics, and immune biomarkers. Findings were synthesized qualitatively according to biomarker category and biological function. **Results:** Functional cellular biomarkers, particularly the basophil activation test, show the greatest translational readiness, with high diagnostic specificity, utility in reaction threshold and severity assessment, and potential value for monitoring oral immunotherapy. Biomarkers of epithelial barrier dysfunction, including zonulin, tight junction proteins, epithelial injury markers, filaggrin variants, and epithelial-derived cytokines, provide mechanistic insight into allergic sensitization and gastrointestinal phenotypes but remain insufficiently validated. Microbiota-derived, metabolomic, transcriptomic, proteomic, epigenetic, and integrated multi-omics approaches offer promising tools for risk prediction, tolerance monitoring, endotype identification, and precision medicine. **Conclusions:** Emerging biomarkers may improve diagnosis, risk stratification, therapeutic monitoring, and personalized care in pediatric food allergy. However, standardized assays, large longitudinal pediatric studies, and external validation are required before routine clinical implementation.

## 1. Introduction

Food allergy is defined as a specific and reproducible immune-mediated adverse reaction to food, with clinical manifestations ranging from mild local symptoms to severe systemic reactions, including anaphylaxis. It represents a major public health concern, particularly because food-induced reactions are among the leading causes of anaphylaxis outside the hospital setting and require accurate diagnosis, risk assessment, and specialized management. Food allergy must be distinguished from food intolerance, which is generally characterized by functional or gastrointestinal symptoms and does not involve an immunological mechanism. For many years, the absence of uniform definitions complicated the estimation of global incidence and prevalence [1]. More recently, the 2023 revision of the European Academy of Allergy and Clinical Immunology nomenclature classified food allergies according to their underlying immunopathogenetic mechanisms, distinguishing between immunoglobulin E (IgE)-mediated forms and cell- and tissue-mediated, non-IgE-mediated forms [2].

Over recent decades, the prevalence of food allergy has increased worldwide, although epidemiological estimates remain influenced by differences in study design, diagnostic criteria, geographic area, and whether data are self-reported or confirmed by oral food challenge. The increase appears more pronounced in industrialized countries, including the United States, and affects children more frequently than adults. Analyses based on self-reported data suggest an estimated rise of up to 1.2% per decade; currently, approximately 8% of children are reported to have food allergy, 2.4% have multiple food allergies, and up to 3% report episodes of anaphylaxis [3]. In Europe, Spolidoro et al. reported that cumulative self-reported lifetime prevalence varies by food allergen, reaching 5.7% for cow’s milk, 2.4% for egg, 1.6% for wheat, 0.5% for soy, 1.5% for peanut, 0.9% for tree nuts, 1.4% for fish, and 0.4% for crustaceans. However, prevalence estimates are markedly lower when diagnosis is confirmed by oral food challenge, ranging from 0.02% for fish to 0.8% for egg [4].

IgE-mediated food allergy is a prototypical type I hypersensitivity reaction characterized by an immediate immune response to specific food allergens. In this process, T cells contribute to B-cell activation and class switching, leading to the production of allergen-specific IgE, which plays a central role in allergic sensitization and effector responses [5]. Clinically, allergies to cow’s milk, egg, soy, and wheat usually arise in early childhood and often resolve before adolescence, whereas allergies to tree nuts, fish, and crustaceans tend to develop later and more frequently persist into adulthood [6]. Non-IgE-mediated food allergies comprise a heterogeneous group of immune reactions independent of IgE, mainly driven by cell-mediated mechanisms, particularly type IVb and V hypersensitivity, and characterized by prominent tissue involvement [7]. Their pathogenesis involves T lymphocytes, eosinophils, and a complex cytokine network [8]. The main clinical entities include food protein-induced enterocolitis syndrome (FPIES), food protein-induced allergic proctocolitis (FPIAP), and food protein-induced enteropathy (FPE) [7]. FPIAP is the most frequently described form, although its true prevalence remains uncertain because of marked variability in epidemiological data [9,10,11]. FPE is rare and appears to be decreasing over time [9]. FPIES, previously considered uncommon, is now estimated to have a cumulative incidence of approximately 0.3–0.7% during the first year of life, with differences across studies likely reflecting geographic and methodological factors [12,13,14]. Mixed forms of food allergy also occur, in which IgE-mediated and non-IgE-mediated mechanisms coexist. These include eosinophilic gastrointestinal diseases, in which the immune response is complex, the contribution of IgE is variable, and clinical manifestations depend on the gastrointestinal segment involved and the degree of eosinophilic infiltration [9].

Accurate diagnosis is essential to guide appropriate dietary, therapeutic, and educational strategies and to prevent severe, potentially life-threatening reactions. At the same time, correct identification of tolerance is crucial to avoid unnecessary dietary restrictions, particularly in light of current preventive approaches supporting the early introduction of potentially allergenic foods [5]. Diagnostic evaluation is based on clinical history and physical examination, supported by evidence of sensitization through skin prick testing (SPT) and/or measurement of serum specific IgE (sIgE) directed against the suspected food allergen [15]. In IgE-mediated food allergy, clinical history remains the cornerstone of the diagnostic work-up but is insufficient on its own to confirm the diagnosis. Confirmation may require a double-blind, placebo-controlled food challenge (DBPCFC), which yields positive results in approximately 30–40% of suspected cases [16]. Key diagnostic elements include the type of symptoms, their temporal relationship with food ingestion, usually within minutes to 1–2 h, reproducibility after subsequent exposures, the form and amount of food ingested, and exclusion of alternative conditions that may mimic food allergy. Clinical assessment should also consider cofactors such as febrile infections, asthma exacerbations, physical exercise, alcohol intake, and medications, including antacids and nonsteroidal anti-inflammatory drugs, which may increase reaction severity [17].

SPT using commercial allergen extracts is a rapid, simple, and reproducible in vivo method for detecting food-specific IgE bound to cutaneous mast cells [16]. Test reactivity may vary according to age, anatomical site, testing device, allergen extract characteristics, and the use of fresh food [17]. Although SPT has limited positive predictive value, its high negative predictive value, exceeding 90%, makes it particularly useful for excluding food allergy in the appropriate clinical context [16,17]. Measurement of sIgE is also widely used because of its high sensitivity, although specificity is limited. Detectable sIgE alone does not confirm clinical allergy, even though higher levels are generally associated with a greater likelihood of reactivity. Conversely, 10–25% of patients may experience reactions despite undetectable IgE levels [17]. When the diagnosis remains uncertain, confirmation may require a medically supervised oral food challenge (OFC), which may be performed as an open-label, single-blind, or double-blind placebo-controlled food challenge depending on the clinical context; among these, the DBPCFC is considered the most rigorous diagnostic format [16,18]. Absence of symptoms during the procedure defines a negative test and allows reintroduction of the food, whereas objective or persistent symptoms confirm a positive response and the diagnosis of food allergy [16].

**Figure 1:**
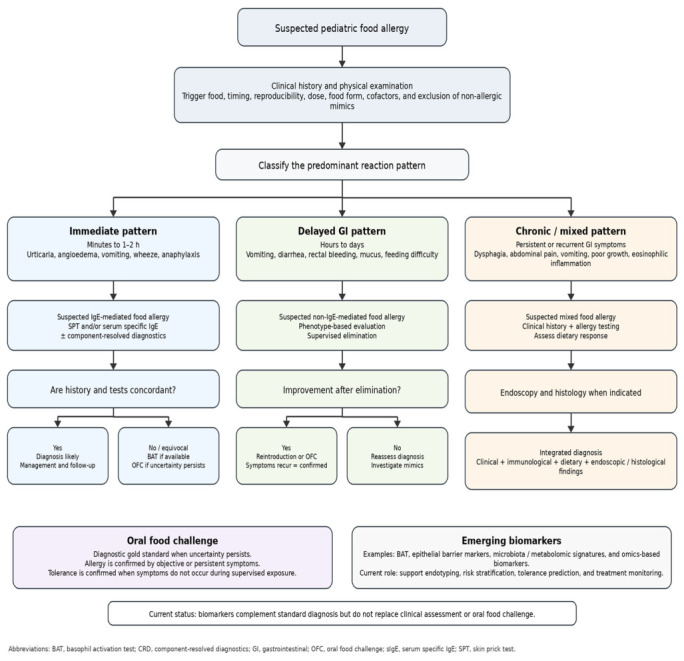
Diagnostic algorithm for suspected pediatric food allergy.

Diagnosis of non-IgE-mediated and mixed food allergies is more challenging. Compared with IgE-mediated forms, symptoms usually occur later after ingestion and may follow a chronic or relapsing course, making the association with the causative food less evident [19]. In addition, the lack of reliable laboratory tests limits instrumental support. In clinical practice, diagnosis relies on recognition of a compatible clinical phenotype, symptom improvement during elimination of the suspected food, and recurrence after reintroduction. OFC remains the diagnostic gold standard also in these forms. In selected cases of mixed food allergy, particularly eosinophilic gastrointestinal diseases, endoscopic assessment may help demonstrate eosinophilic infiltration of the gastrointestinal mucosa [19]. To provide a clearer overview of the diagnostic framework, the conventional diagnostic pathway for suspected pediatric food allergy is summarized in Figure 1.

Serum sIgE measurement represents one of the cornerstone tests in the current diagnostic work-up of IgE-mediated food allergy and is widely used together with clinical history and SPT to assess allergic sensitization. However, detectable sIgE indicates sensitization rather than clinical allergy, and its interpretation requires careful correlation with the patient’s symptoms, timing of reactions, culprit food, age, and likelihood of natural tolerance acquisition [20]. Although higher sIgE concentrations are generally associated with an increased probability of clinical reactivity, sIgE alone has limited specificity and does not reliably predict reaction severity or individual threshold dose. For this reason, sIgE is discussed in this review as part of the established diagnostic framework rather than as an emerging biomarker. The focus of the present review is instead on novel or evolving biomarkers that may complement conventional testing by improving diagnostic specificity, supporting endotype identification, predicting severity or reaction thresholds, monitoring tolerance acquisition, or assessing response to therapies. In this context, newer approaches such as component-resolved diagnostics and functional assays, particularly the basophil activation test, may provide information beyond conventional extract-based sIgE measurement and are therefore considered in greater detail [19].

Within this diagnostic and clinical framework, the identification of reliable biomarkers capable of predicting the risk of food allergy development, reaction severity, persistence, tolerance acquisition, or response to therapy would represent a major advance. Such biomarkers could support earlier intervention, reduce food allergy–related morbidity and mortality, limit the need for invasive or high-risk diagnostic procedures, and help identify novel therapeutic targets [20]. Biomarkers able to define specific phenotypes and endotypes are also essential to improve diagnostic accuracy and personalize treatment strategies. At present, management remains largely based on patient and caregiver education, allergen avoidance, and prompt treatment of accidental reactions [21]. However, advances in immunology, high-throughput technologies, and bioinformatics have led to the identification of several promising biomarker categories in food allergy [22].

Despite the increasing number of studies in this field, the integration of biomarkers into routine clinical practice remains limited. Current evidence is characterized by substantial heterogeneity in study design, patient populations, laboratory methods, and clinical outcomes [19,20,21,22]. Many candidate biomarkers lack standardized cut-off values, external validation, longitudinal pediatric data, and clear evidence of clinical utility. Moreover, although several reviews have addressed emerging biomarkers in food allergy, few have critically evaluated their diagnostic performance, biological relevance, translational readiness, and potential applicability within precision medicine frameworks, particularly in pediatric populations [19,20,21,22].

For these reasons, the present narrative review aims to provide a comprehensive and critical evaluation of emerging biomarkers in pediatric food allergy, focusing on their diagnostic, prognostic, predictive, and therapeutic potential. Particular attention is given to biomarkers supported by increasing clinical and translational evidence and capable of reflecting distinct pathophysiological mechanisms involved in disease development and progression. The biomarker categories considered in this review include functional cellular biomarkers, such as the basophil activation test (BAT), which may improve diagnostic specificity, risk stratification, and reduce the need for OFCs; biomarkers of epithelial barrier dysfunction and intestinal permeability, which may help characterize gastrointestinal and non-IgE-mediated phenotypes; microbiota-derived and metabolomic biomarkers, which are increasingly linked to oral tolerance, immune regulation, and disease persistence; and inflammatory, transcriptomic, proteomic, epigenetic, and other immunological biomarkers that may contribute to endotype identification and future personalized therapeutic strategies. An overview of the main biomarker categories, their biological rationale, potential clinical utility, and current validation status is provided in Table 1.

It should be noted that the biomarker evidence base is substantially stronger for IgE-mediated pediatric food allergy than for non-IgE-mediated or mixed phenotypes. Functional and sensitization-based biomarkers, including sIgE, component-resolved diagnostics, and BAT, are mainly applicable to IgE-mediated disease. By contrast, candidate biomarkers for non-IgE-mediated and mixed gastrointestinal food allergies include epithelial barrier markers, fecal inflammatory mediators, eosinophil-associated proteins, epithelial cytokines, microbiota-derived and metabolomic signatures, and omics-based profiles, but these remain largely exploratory and insufficiently validated. This review therefore distinguishes, where possible, between biomarkers with evidence in IgE-mediated disease and those proposed for non-IgE-mediated or mixed phenotypes.

By analyzing these biomarker categories, this review aims not only to summarize current evidence but also to identify the main barriers preventing implementation in routine clinical practice, including assay standardization, reproducibility, accessibility, methodological variability, and lack of prospective validation. Ultimately, this review seeks to clarify which biomarkers currently show the strongest translational potential and which remain investigational, thereby supporting the development of precision medicine approaches in pediatric food allergy. Although the diagnostic tools discussed in this review, including clinical history, physical examination, SPT, serum specific IgE measurement, component-resolved diagnostics, BAT, and OFC, are not exclusive to children and may be applied across all age groups, this review specifically focuses on pediatric food allergy because disease onset, immunological maturation, natural history, tolerance acquisition, nutritional consequences, and diagnostic decision-making differ substantially between children and adults. In pediatric patients, diagnostic strategies must account for age-dependent sensitization patterns, evolving immune and epithelial barrier function, the higher likelihood of spontaneous resolution for some food allergies, the impact of elimination diets on growth and development, and the need to minimize invasive or high-risk procedures whenever possible. Therefore, the term “pediatric diagnostics” is used here not to indicate tests unique to children, but to emphasize the pediatric clinical context in which these universal diagnostic tools and emerging biomarkers are interpreted and applied. Although several reviews have addressed emerging biomarkers in food allergy, fewer have focused specifically on pediatric populations while simultaneously assessing biological plausibility, available diagnostic and prognostic performance, assay reproducibility, standardization, external validation, clinical accessibility, and translational readiness within precision-medicine frameworks. In this review, the term “endotyping” refers to the attempt to classify pediatric food allergy according to underlying biological mechanisms rather than clinical presentation alone. Potential endotypes may include, for example, IgE-mediated effector-cell activation, epithelial barrier dysfunction, altered host–microbiota metabolism, type 2 inflammatory activation, or immune-regulatory/tolerance-associated pathways. However, most biomarkers discussed in this review should not yet be considered definitive endotype-defining tools. Rather, they currently provide mechanistic signals that may help characterize disease heterogeneity and support future endotype-based classification once validated in longitudinal pediatric cohorts. Therefore, the expression “mechanistic endotyping” is used here to indicate the use of biomarkers to explore biologically meaningful disease subgroups, while acknowledging that only a limited number of candidate markers have sufficient evidence for clinical endotype assignment at present.

## 2. Methods

This narrative review was supported by a structured literature search designed to identify relevant clinical, translational, and mechanistic evidence on emerging biomarkers in pediatric food allergy. The search was conducted in PubMed/MEDLINE and the Cochrane Central databases, with priority given to articles published between December 2015 and March 2026. Additional landmark studies, guideline documents, and background publications published outside this time frame were also considered when they provided important clinical, mechanistic, or methodological context.

The search strategy combined terms related to food allergy, biomarkers, and pediatric populations, including “food allergy,” “biomarkers,” “markers,” “children,” “child,” “infant,” “toddler,” “adolescent,” “pediatric,” and “paediatric.” Additional targeted searches were performed for specific biomarker categories, including “basophil activation test,” “epithelial barrier biomarkers,” “intestinal permeability,” “zonulin,” “filaggrin,” “gut microbiota,” “short-chain fatty acids,” “metabolomics,” “transcriptomics,” “proteomics,” “epigenetic biomarkers,” “microRNA,” “inflammatory biomarkers,” and “immune biomarkers.”

Peer-reviewed articles published in English were prioritized. Publications were considered for inclusion if they addressed pediatric food allergy directly or provided mechanistic, diagnostic, prognostic, predictive, or therapeutic information applicable to biomarker research in pediatric food allergy. Particular attention was given to studies evaluating biomarker performance, biological plausibility, association with disease phenotype or endotype, prediction of reaction severity or threshold, tolerance acquisition, and monitoring of response to therapeutic interventions.

Overall, 127 publications were ultimately analyzed and included in the narrative synthesis. These comprised original clinical and translational studies, mechanistic investigations, guideline documents, and selected reviews considered relevant to biomarker development, diagnostic evaluation, disease endotyping, tolerance prediction, therapeutic monitoring, and precision-medicine approaches in pediatric food allergy.

Because this article was designed as a narrative review rather than a systematic review, no formal systematic review protocol was registered, and no standardized risk-of-bias assessment or meta-analysis was performed. Study selection was based on relevance to the review objectives, methodological quality, clinical applicability, and contribution to understanding the role of biomarkers in pediatric food allergy. Findings were synthesized qualitatively and organized according to biomarker category and biological function, including functional cellular biomarkers, epithelial barrier and intestinal permeability biomarkers, microbiota-derived and metabolomic biomarkers, inflammatory and immunological biomarkers, and omics-based approaches.

The aim of this methodological approach was to provide a comprehensive and clinically oriented overview of emerging biomarkers in pediatric food allergy, while critically discussing their biological rationale, current level of validation, translational readiness, and potential role in precision medicine.

## 3. Cellular and Functional Biomarkers

The BAT is a flow cytometry–based functional assay that measures the upregulation of activation markers, most commonly CD63 and CD203c, on the surface of basophils after stimulation with specific allergens. Since its development, BAT has progressively gained relevance in the diagnosis and monitoring of allergic diseases, emerging as a safe, reproducible, and informative in vitro alternative to in vivo provocation tests. Because it is performed entirely *ex vivo*, BAT is less invasive and safer than OFC, while also offering potential cost advantages in selected diagnostic pathways [23,24,25,26,27,28,29,30,31,32,33,34,35,36,37,38,39,40].

In the 2024 update of the European Academy of Allergy and Clinical Immunology (EAACI) guidelines on the diagnosis of IgE-mediated food allergy, BAT was included among recommended diagnostic tools for the first time. It is suggested as a second-line test in patients with suspected food allergy and equivocal results on SPT and/or serum sIgE, before proceeding to OFC [41]. This recommendation reflects the growing evidence supporting the diagnostic accuracy of BAT, particularly for peanut and sesame allergy, for which meta-analyses have shown moderate sensitivity, 86% and 89%, respectively, and high specificity, 90% and 93%, respectively, with low heterogeneity across studies [42].

The biological rationale for BAT is closely linked to the effector mechanisms of IgE-mediated food allergy. Acute allergic reactions and anaphylaxis result from activation and degranulation of mast cells and basophils after allergen-induced cross-linking of IgE bound to the high-affinity IgE receptor FcεRI. Basophils express the tetrameric form of FcεRI and, after allergen stimulation, release vasoactive and inflammatory mediators that contribute directly to acute allergic symptoms. Unlike mast cells, basophils are readily accessible in peripheral blood, making them particularly suitable for functional diagnostic testing [23,24,25,26,27,28,29,30,31,32,33,34,35,36,37,38,39,40].

BAT evaluates basophil activation after stimulation with allergens or control stimuli. Activated basophils upregulate surface proteins that can be quantified by flow cytometry. CD63, a lysosome-associated four-transmembrane protein localized in secretory granules, translocates to the plasma membrane during degranulation and is the most widely used activation marker. CD203c is also frequently assessed and is commonly expressed as a stimulation index [23,43]. Basophil activation increases with rising allergen concentrations until a plateau is reached. The resulting dose–response curve is influenced by several factors, including the density of epitope–IgE complexes, IgE affinity for the allergen, and intrinsic basophil responsiveness [44]. Therefore, BAT optimization requires allergen dose–response curves using multiple, usually 6–8, serial dilutions to identify the most informative diagnostic concentration [45,46]. For each food allergen, specific cut-off values for basophil activation, expressed as the percentage of CD63-positive basophils and/or CD203c upregulation, should be defined to maximize diagnostic accuracy, including area under the curve (AUC), specificity, and sensitivity [45].

Methodological standardization is essential for reliable BAT interpretation. Negative controls usually show 1.5–2.5% spontaneous basophil activation; therefore, 2.5% CD63-positive basophils is generally accepted as the upper limit for valid negative controls [47,48]. Positive controls should be included in every assay and commonly consist of anti-IgE, anti-FcεRI antibodies, or N-formyl-methionyl-leucyl-phenylalanine (fMLP). Anti-IgE and anti-FcεRI induce IgE-dependent activation through receptor cross-linking, whereas fMLP activates basophils through G-protein–coupled receptors in an IgE-independent manner [49,50,51]. BAT requires less than 0.1 mL of fresh blood and should ideally be performed within 4 h of sampling, and no later than 24 h, because basophil responsiveness declines rapidly over time [52,53,54,55].

In food allergy, BAT provides substantially higher specificity than SPT and sIgE, which are sensitive but often limited by poor specificity and may leave patients in a diagnostic “grey zone.” BAT has demonstrated specificity up to 100% for peanut allergy [43,56] and egg allergy [57], and has been useful in distinguishing allergic from tolerant children sensitized to milk or egg, including children with atopic dermatitis [58,59]. Consequently, BAT may reduce the need for diagnostic OFC in patients with equivocal first-line test results.

This advantage is particularly relevant in polysensitized children with suspected tree nut and seed allergy. In a large prospective European multicenter study, 60.7% of children with challenge-confirmed peanut, tree nut, or sesame allergy were allergic to more than one nut or seed, highlighting the difficulty of interpreting multiple positive SPT and sIgE results for foods not regularly consumed [50]. Although sensitization to multiple nuts is common, many patients react clinically to only one or two, with strong cross-reactivity observed between cashew and pistachio and between walnut and pecan [50,51]. In this context, BAT used as a second-line diagnostic tool has been shown to reduce the need for OFC by 5–15% and the number of positive OFCs by 33–75%, while achieving diagnostic accuracy between 96% and 100% and no false-negative results [60].

BAT may also support diagnostic decision-making in cow’s milk and egg allergy, the most common food allergies worldwide [61,62]. Approximately 60–80% of affected patients tolerate these foods in baked form, allowing dietary liberalization and improving quality of life [63]. Baked milk and egg may also be incorporated into oral immunotherapy (OIT) protocols and may accelerate the natural resolution of allergy [63,64]. Because SPT and sIgE poorly predict baked food tolerance, OFC is often required. In this setting, BAT has emerged as a useful tool for selecting patients for challenge. BAT has shown particular utility in predicting baked egg allergy in young children and, when combined with sIgE, has been associated with a 30% reduction in OFCs [65]. Increased basophil activation to milk has also been associated with reactivity and severity during baked milk challenges [66].

Beyond diagnosis, BAT provides information on reaction severity and clinical thresholds. Basophil reactivity, usually expressed as the percentage of CD63-positive basophils, correlates with reaction severity, whereas basophil sensitivity parameters, such as CD-sens or EC50, correlate with clinical threshold doses [56,67,68]. These findings have been confirmed in large pediatric cohorts, including participants in the LEAP study. Increased basophil activation has also been associated with severe reactions and epinephrine use in walnut allergy [69], and BAT has emerged as one of the most accurate biomarkers for predicting severity and low reaction thresholds in egg allergy during double-blind placebo-controlled food challenges [70].

BAT may also have a role in therapeutic stratification and monitoring. In patients undergoing OIT or receiving biologics targeting T helper 2 (Th2) inflammation, such as omalizumab, elevated basophil activation has been proposed as a biomarker to identify candidates who may benefit from treatment and to monitor immunological changes over time [71]. Omalizumab induces complex effects on basophils, including reduced FcεRI density and increased intrinsic basophil sensitivity, changes that can be detected by BAT and correlated with clinical outcomes [72,73].

Despite its strong diagnostic performance, BAT remains a second-line test because of limited availability, higher costs compared with SPT and sIgE, and the need for standardized protocols, fresh blood samples, flow cytometry infrastructure, and trained personnel. In addition, approximately 10% of individuals have basophils that do not respond to FcεRI-mediated stimulation, making BAT results uninterpretable in these cases [57]. Furthermore, although evidence is robust for peanut and sesame allergy, BAT has not yet been formally recommended by EAACI for all foods because of insufficient data for meta-analysis.

Overall, BAT represents the most clinically advanced functional biomarker in pediatric food allergy. Its main diagnostic and prognostic features, including activation markers, sample requirements, sensitivity, specificity, ability to reduce OFCs, and utility for severity, threshold, and treatment monitoring, are summarized in Table 2.

Wider clinical implementation will require further standardization, external quality assurance, and improved accessibility, but the potential impact of BAT on precision diagnosis and risk stratification in pediatric food allergy is substantial.

## 4. Inflammatory and Immunological Biomarkers

### 4.1. Biomarkers of Intestinal Barrier Permeability

The intestinal epithelial barrier plays a central role in maintaining immune homeostasis and oral tolerance to dietary antigens. It consists of a single layer of epithelial cells interconnected by tight junctions, adherens junctions, and gap junctions, which regulate the selective passage of molecules through the paracellular pathway. Tight junctions are the principal regulators of intestinal permeability and are composed of transmembrane proteins, including claudins, occludin, junctional adhesion molecules, and tricellulin. These proteins interact with cytoplasmic scaffold proteins of the zonula occludens family, particularly ZO-1, ZO-2, and ZO-3, which connect the junctional complex to the actin cytoskeleton. Under physiological conditions, this barrier limits the systemic passage of intact food antigens while allowing nutrient absorption and controlled immune sampling. Disruption of epithelial integrity increases intestinal permeability, facilitates allergen translocation into the lamina propria, and promotes immune activation and allergic sensitization [74].

Growing evidence suggests that epithelial barrier dysfunction is a key pathogenic mechanism in food allergy and may define a specific disease endotype characterized by enhanced mucosal permeability, impaired barrier regulation, and altered immune responses. Accordingly, several biomarkers reflecting intestinal barrier integrity, epithelial injury, microbial translocation, and mucosal inflammation have been investigated as potential tools for disease characterization, risk stratification, and monitoring in pediatric food allergy [75].

Tight junction-related biomarkers reflect structural or functional alterations of epithelial junctional complexes and include zonulin, claudins, occludin, and zonula occludens proteins, particularly ZO-1. Among these, zonulin is currently the most extensively investigated biomarker in food allergy because of its role in modulating tight junction permeability and regulating paracellular antigen passage [76,77]. Alterations in tight junction–associated proteins may indicate impaired epithelial integrity and increased exposure of the mucosal immune system to food antigens.

Enterocyte damage biomarkers indicate epithelial cell injury and mucosal damage. The most commonly investigated markers include intestinal fatty acid-binding protein (I-FABP) and diamine oxidase (DAO). Increased circulating levels of these molecules may reflect enterocyte injury, impaired mucosal integrity, and increased intestinal permeability, although evidence in pediatric food allergy remains limited and requires further validation [78,79].

Microbial translocation and systemic permeability markers indirectly reflect enhanced passage of luminal microbial products across a compromised intestinal barrier. These include lipopolysaccharide-binding protein (LBP), soluble CD14, and circulating endotoxin-related markers. Such biomarkers have mainly been studied in experimental settings and systemic inflammatory conditions and may provide mechanistic information on the relationship between barrier disruption, microbial exposure, and immune activation [79,80].

Fecal inflammatory and mucosal immunity biomarkers are not specific markers of intestinal permeability but may reflect mucosal inflammation associated with barrier dysfunction. These include fecal calprotectin, secretory IgA, and eosinophil-derived proteins, such as eosinophilic cationic protein (ECP) and eosinophil-derived neurotoxin (EDN). Alterations in these markers have been reported in pediatric food allergy, particularly in children with gastrointestinal involvement, non-IgE-mediated phenotypes, and eosinophilic inflammation [75].

Overall, biomarkers of epithelial barrier dysfunction may contribute to the identification of specific food allergy phenotypes, especially in patients with gastrointestinal manifestations and non-IgE-mediated disease. However, most remain investigational because of limited assay standardization, heterogeneity among studies, small pediatric cohorts, and insufficient longitudinal validation. At present, no intestinal permeability biomarker has been fully validated for routine clinical diagnosis of food allergy, although several candidates show promise for future endotype-based and precision medicine approaches [21].

### 4.2. Epithelial Barrier Dysfunction and Integrity Biomarkers in Allergic Sensitization

According to the epithelial barrier hypothesis, impairment of epithelial surfaces represents a central and early mechanism in the pathogenesis of allergic diseases, including food allergy [75]. Increasing evidence suggests that intestinal and cutaneous barrier dysfunction may contribute causally to allergic sensitization rather than merely reflecting secondary inflammation [74]. Disruption of epithelial integrity can increase antigen passage across epithelial surfaces, promote immune activation, impair tolerogenic pathways, and favor type 2 inflammatory responses.

Clinical observations support this concept. Infants with early-onset atopic dermatitis (AD) are at increased risk of developing food allergy later in childhood. Population-based studies have further demonstrated that eczema and loss-of-function mutations in the filaggrin (FLG) gene, which impair skin barrier integrity, are major risk factors for allergic sensitization and subsequent food allergy [81,82]. These findings support an integrated epithelial barrier model in which both cutaneous and intestinal barrier dysfunction may promote allergen exposure, immune activation, and progression toward clinically relevant food allergy.

Experimental data further indicate that the transition from skin sensitization to intestinal allergic inflammation is a critical step in the development of IgE-mediated food allergy. In models of epicutaneous sensitization with thymic stromal lymphopoietin (TSLP) and ovalbumin, blockade of TSLP alone does not completely prevent food allergy development, suggesting that additional intestinal immune pathways contribute to disease amplification [83]. The gastrointestinal mucosa is the largest immune interface exposed to dietary antigens. Under physiological conditions, CD103^+^ tolerogenic dendritic cells promote oral tolerance by inducing antigen-specific regulatory T cells. However, disruption of epithelial integrity, increased antigen passage, and altered epithelial cytokine signaling may impair these tolerogenic pathways and favor type 2 immune responses [84].

In this context, biomarkers associated with epithelial barrier integrity may help identify children with barrier-impaired phenotypes or endotype-oriented patterns. These include structural epithelial proteins, tight junction-associated molecules, epithelial-derived cytokines, and genetic variants involved in barrier formation and maintenance [85,86,87,88,89]. Unlike circulating inflammatory mediators, epithelial integrity markers may reflect primary defects in epithelial structure or function and may therefore provide mechanistic information on susceptibility to allergic sensitization, disease persistence, and clinical severity [88].

Among epithelial-derived cytokines, interleukin-25 (IL-25) has emerged as a potential mediator linking epithelial dysfunction to intestinal allergic inflammation. IL-25, constitutively produced by tuft cells, promotes type 2 immunity through activation of group 2 innate lymphoid cells and enhanced production of interleukin-5 (IL-5) and interleukin-13 (IL-13). Experimental studies have shown that increased IL-25 signaling enhances susceptibility to food allergy, whereas deletion of its receptor confers protection in animal models [84]. Other epithelial alarmins involved in barrier dysfunction, including TSLP and interleukin-33 (IL-33), are also promising candidates for endotype characterization but currently lack validated cut-off values, standardized assays, and longitudinal pediatric data supporting routine clinical use [21,22].

Overall, epithelial barrier dysfunction should be viewed both as a potential early pathogenic event in allergic sensitization and as a source of candidate biomarkers for endotype-oriented stratification. However, most epithelial integrity biomarkers remain investigational because of limited pediatric validation, lack of standardized assays, absence of validated cut-off values, and insufficient longitudinal evidence. Future studies using standardized methods, clearly defined pediatric phenotypes, and prospective longitudinal designs are required before these markers can be incorporated into routine clinical practice.

### 4.3. Zonulin as a Biomarker of Intestinal Permeability

Among biomarkers of intestinal barrier dysfunction, zonulin has received considerable attention in food allergy and other immune-mediated gastrointestinal disorders. Its biological appeal derives from its proposed role as a physiological modulator of intercellular tight junctions and intestinal permeability. Increased zonulin signaling has been associated with tight junction disassembly and enhanced paracellular trafficking of luminal antigens and microbial products across the intestinal epithelium, mechanisms that are highly relevant to allergic sensitization and mucosal immune activation [76,77]. However, zonulin also represents an important cautionary example of how promising biological rationale and early clinical associations may not be sufficient for biomarker translation when analytical validity remains uncertain.

Several pediatric studies have reported elevated serum zonulin levels in allergic conditions. In a study evaluating selected intestinal permeability markers in children with food allergy, serum zonulin concentrations were significantly higher than in healthy controls and were particularly increased in patients with gastrointestinal manifestations and non-IgE-mediated food allergy [75]. Additional evidence comes from pediatric AD, a condition closely associated with food sensitization and food allergy. Sheen et al. reported that serum zonulin levels were significantly associated with both the presence and severity of atopic dermatitis in children, independently of total IgE and eosinophil counts, supporting the hypothesis that epithelial barrier dysfunction may contribute to allergic inflammation beyond classical IgE-mediated mechanisms [85]. These findings suggest that increased intestinal permeability may coexist with impaired epithelial barrier integrity and chronic allergic inflammation in food allergy and related atopic disorders.

### 4.4. Filaggrin: Linking Skin Barrier Dysfunction to Food Allergy

Filaggrin is a key structural protein involved in epidermal differentiation and maintenance of skin barrier integrity. It contributes to keratin aggregation, epidermal hydration, and prevention of transepidermal water loss. Loss-of-function mutations in the FLG gene impair skin barrier function, facilitating allergen penetration through the epidermis and promoting transcutaneous sensitization [90]. The association between FLG mutations and AD is well established, and accumulating evidence indicates that impaired skin barrier integrity may represent an early step in the allergic march leading to food allergy and asthma [89,91].

Several studies have demonstrated that children carrying *FLG* loss-of-function variants are at increased risk of developing peanut allergy and other IgE-mediated food allergies, particularly in the presence of early-onset AD [92]. In addition to increasing susceptibility, *FLG* mutations have been associated with greater disease severity and persistence. Astolfi et al. reported that *FLG* loss-of-function mutations were significantly associated with severe food allergy in children with atopic dermatitis, supporting the hypothesis that genetically impaired epithelial barriers may contribute not only to sensitization but also to more severe clinical phenotypes [93].

Despite their pathogenic and prognostic relevance, *FLG* mutations should be considered susceptibility biomarkers rather than dynamic disease biomarkers. Unlike circulating inflammatory mediators or markers of epithelial permeability, *FLG* variants are genetically determined and remain stable over time. Therefore, they are more useful for identifying children at increased risk of allergic sensitization, persistent disease, or severe phenotypes than for monitoring disease activity or therapeutic response in clinical practice [21].

Overall, *FLG* mutations provide important evidence supporting epithelial barrier dysfunction as a central mechanism in food allergy pathogenesis. Their current clinical relevance lies mainly in risk stratification and in the identification of barrier dysfunction-associated allergic endotypes, particularly in pediatric patients with early-onset atopic dermatitis.

## 5. Gut Microbiota as a Potential Biomarker in Pediatric Food Allergies

The gut microbiota has emerged as a promising source of candidate biomarkers in pediatric food allergy because of its central role in oral tolerance, epithelial barrier integrity, and immune regulation. Early-life microbial dysbiosis has been repeatedly associated with increased susceptibility to food allergy, supporting the hypothesis that microbiota-related signatures may contribute to disease prediction, endotype definition, and monitoring of tolerance acquisition [93,94,95]. However, despite growing evidence, microbiota-derived biomarkers remain largely exploratory and are not yet standardized for clinical use.

Microbiota-derived biomarkers in food allergy can be broadly classified into four overlapping categories: compositional biomarkers, including bacterial taxa and microbial diversity patterns; metabolomic biomarkers, such as short-chain fatty acids (SCFAs), bile acids, and tryptophan-derived metabolites; functional microbial pathways involved in immune regulation and epithelial integrity; and integrated multi-omics signatures combining metagenomics, metabolomics, transcriptomics, and immunophenotyping data. Rather than representing a single biomarker, the gut microbiota should therefore be considered a multidimensional biomarker platform with potential relevance for disease stratification and precision medicine.

Several studies have identified reduced microbial diversity and depletion of specific commensal taxa in children with food allergy. In particular, lower abundances of *Clostridiales, Bifidobacterium*, and butyrate-producing bacteria have been associated with impaired oral tolerance and increased allergic sensitization [96,97]. These alterations may reduce regulatory T-cell (Treg) induction, weaken epithelial barrier function, and favor T helper 2 (Th2)-skewed immune responses. Nevertheless, most available evidence remains associative rather than causal. Moreover, compositional microbial signatures show substantial inter-study variability because of differences in age, diet, geographic setting, breastfeeding, antibiotic exposure, sequencing methodology, and bioinformatic pipelines. These factors limit inter-cohort reproducibility and currently reduce the reliability of microbiota composition as a standalone diagnostic biomarker.

Among microbiota-derived candidates, SCFAs, particularly butyrate, are among the most extensively investigated functional biomarkers. SCFAs contribute to epithelial barrier maintenance, mucus production, peripheral Treg differentiation, and immune tolerance [95,97,98]. Reduced fecal butyrate concentrations have been associated with food allergy and persistent cow’s milk allergy, suggesting a possible prognostic role in tolerance acquisition [98]. In longitudinal pediatric cohorts, lower fecal butyrate levels during infancy have been linked to an increased risk of persistent allergic phenotypes, with some studies reporting moderate discriminatory performance for tolerance prediction. However, SCFA concentrations are strongly influenced by diet, age, sample collection and storage, and analytical methodology. Validated cut-off values and externally validated sensitivity and specificity thresholds are still lacking, preventing routine clinical application.

Only a limited number of studies have evaluated microbiota-derived biomarkers using robust quantitative performance metrics. Available evidence suggests that integrated microbiota–metabolomic models generally outperform single microbial taxa, showing moderate-to-good discriminatory capacity in differentiating allergic from non-allergic children, with area under the curve values varying according to the cohort and omics platform used [99]. Similarly, some longitudinal studies suggest that early-life microbial and metabolomic signatures may predict food allergy persistence or tolerance acquisition, supporting their potential value as dynamic prognostic biomarkers [95,100]. However, sensitivity, specificity, positive predictive value, and hazard ratio estimates are inconsistently reported, and few candidate biomarkers have been externally validated in independent pediatric cohorts. Consequently, current evidence remains insufficient to define clinically applicable diagnostic thresholds or standardized predictive models.

Beyond SCFAs, increasing attention has focused on other metabolomic signatures, including secondary bile acids and tryptophan-derived metabolites. Altered bile acid metabolism has been associated with food allergy development and may reflect disrupted microbial metabolic activity and altered mucosal immune signaling [95]. Similarly, perturbations in tryptophan metabolism may affect aryl hydrocarbon receptor (AhR)-mediated immune regulation and epithelial homeostasis, supporting their potential role as biomarkers of immune dysregulation in food allergy [98]. Preliminary metabolomic studies have shown promising discriminatory accuracy in differentiating allergic from non-allergic children, although reproducibility across independent pediatric cohorts remains limited. These findings therefore require confirmation in larger, longitudinal, multicenter studies before clinical implementation.

Recent multi-omics approaches integrating metagenomics, metabolomics, transcriptomics, and immunophenotyping have further expanded the biomarker landscape. De Paepe et al. identified integrated microbiota–metabolome signatures capable of discriminating children with food allergy from healthy controls, with improved predictive performance compared with individual microbial taxa alone [99]. Additional studies have reported associations between IgA-coated bacteria and allergic phenotypes, suggesting that host–microbiota immune interactions may provide novel stratification biomarkers [93]. Integrated multi-omics models may ultimately improve disease classification and prediction compared with single biomarkers, although few candidate signatures have undergone external validation in large pediatric longitudinal cohorts.

Importantly, microbiota-related biomarkers may have different clinical applications. Dysbiosis patterns and metabolomic alterations could serve as susceptibility biomarkers for early food allergy risk prediction, whereas SCFAs and functional microbial pathways may provide prognostic information on disease persistence or tolerance acquisition. Multi-omics signatures and immune–microbiota interaction profiles may instead support disease endotyping and patient stratification within precision medicine frameworks.

Despite these promising findings, several limitations currently prevent translation into routine practice. Most studies are characterized by small sample sizes, cross-sectional designs, heterogeneous populations, and lack of external validation. Major methodological variability also exists in stool collection procedures, sequencing technologies, 16S rRNA sequencing versus shotgun metagenomics, metabolomic platforms, and bioinformatic analyses, resulting in inconsistent findings and limited reproducibility. In addition, diet, age, antibiotic exposure, breastfeeding, environmental factors, and geography strongly influence microbiota composition and metabolite profiles, further complicating interpretation and limiting generalizability.

Overall, the gut microbiota represents a highly promising but still exploratory biomarker platform in pediatric food allergy. Future research should prioritize longitudinal multicenter cohorts, standardized sample collection and analytical pipelines, quantitative validation metrics, and integrated multi-omics approaches to define the clinical utility, reproducibility, diagnostic performance, and predictive accuracy of microbiota-derived biomarkers. Such advances may support precision medicine strategies aimed at improving early diagnosis, prognostic stratification, tolerance monitoring, and personalized therapeutic interventions in children with food allergy.

## 6. Biomarkers in Emerging Therapies and Precision Medicine

### 6.1. Epigenetics and Gene Regulation

Epigenetic regulation, defined as heritable and reversible changes in gene expression that do not alter the underlying DNA sequence, has emerged as an important dimension of food allergy pathogenesis. Epigenetic mechanisms may help explain how genetic susceptibility, environmental exposures, microbiota-derived signals, dietary factors, and immunological maturation interact during critical windows of immune development. Among these mechanisms, DNA methylation and microRNA-mediated gene regulation are the most extensively investigated in food allergy and are increasingly considered potential tools for disease prediction, endotype characterization, and monitoring of response to immunomodulatory interventions.

#### 6.1.1. DNA Methylation Signatures in Food Allergy

DNA methylation at cytosine–phosphate–guanine (CpG) dinucleotides is one of the best characterized epigenetic modifications in allergic disease. When occurring within gene promoter regions, CpG methylation generally suppresses gene transcription, although its functional effect depends on genomic location, cell type, and immune context. The T helper 2 (Th2)-skewed immune response that characterizes IgE-mediated food allergy is tightly regulated at the epigenetic level. Candidate-gene studies have shown altered methylation of key cytokine loci, including IL4, IL5, IL10, IFNG, and IL12B, in food-allergic individuals compared with healthy controls. In a systematic review including 16 studies, Safar et al. found that epigenomic associations with food allergy were mainly located in genes involved in Th1/Th2 balance, regulatory T-cell (Treg) function, Toll-like receptor signaling, and intestinal barrier integrity. Negative correlations between promoter methylation of IL-4, IL-5, IL-10, and interferon-γ (IFN-γ) and the corresponding circulating cytokine concentrations have also been reported, supporting the expected suppressive effect of promoter hypermethylation on gene expression [101].

A particularly relevant epigenetic target is *FOXP3*, the master transcription factor required for Treg identity and function. Demethylation of *FOXP3* CpG sites, especially within the Treg-specific demethylated region (TSDR), is associated with stable Treg differentiation and the acquisition of immunological tolerance [102]. In a pivotal study by Syed et al., patients with peanut allergy who achieved sustained unresponsiveness after oral immunotherapy (OIT) showed significantly lower *FOXP3* methylation in antigen-induced Tregs than patients who regained clinical sensitivity after peanut avoidance. Importantly, resensitization was accompanied by remethylation of the same CpG sites, indicating that *FOXP3* methylation may dynamically reflect both acquisition and loss of clinical tolerance [103]. Complementary preclinical evidence suggests that combining OIT with the botanical extract B-FAHF-2 may provide greater and more durable protection than OIT alone, with enhanced efficacy associated with favorable epigenetic changes, including IL-4 promoter remethylation and IFN-γ and *FOXP3* promoter demethylation. Final challenge symptom scores were inversely correlated with IL-4 methylation levels, further supporting the potential role of epigenetic marks as pharmacodynamic biomarkers in OIT research [94]. Together, these findings position *FOXP3* methylation as a candidate pharmacoepigenomic biomarker for monitoring OIT-induced tolerance.

Epigenetic alterations may also appear early in life. In a study of infants younger than six months, CpG methylation profiles of IL-4, IL-5, IL-10, IFN-γ, and *FOXP3* differed significantly between allergic infants, defined as those with food allergy and/or atopic dermatitis, and healthy controls, with lower *FOXP3* methylation observed among allergic infants [104]. This finding requires cautious interpretation. Unlike TSDR-focused studies, in which *FOXP3* hypomethylation reflects stable Treg differentiation and tolerance, the study by Gorzkiewicz et al. used whole-blood high-resolution melting polymerase chain reaction (PCR) targeting a CpG amplicon outside the TSDR [104]. Because methylation changes outside the TSDR may have different functional implications, and because whole blood contains mixed cell populations dominated by non-Treg lineages, these results are not directly comparable with analyses performed in sorted T-cell subsets. Nevertheless, they suggest that disease-associated epigenetic marks may emerge during the first months of life and could contribute to early risk stratification.

At the genome-wide level, epigenome-wide association studies (EWASs) have identified 11 differentially methylated *loci* replicated in at least two independent food allergy studies, including genes involved in T-cell development, antigen presentation, and chromatin remodeling, such as *RPS6KA2, HDAC4, CAMTA1,* and *DOCK1* [101]. More recently, machine learning integration of whole-genome DNA methylation array data identified *LDHC* and *SLC35G2* as candidate epigenetic biomarkers for food allergy classification, with validation across two independent cohorts [103,104,105]. These findings illustrate the growing convergence of epigenomics, computational modeling, and precision medicine.

Overall, DNA methylation studies provide strong biological plausibility for the development of diagnostic, prognostic, and treatment-monitoring biomarkers in food allergy. However, clinical translation remains limited by substantial methodological heterogeneity, including differences in tissue source, such as whole blood, peripheral blood mononuclear cells, and sorted T-cell populations; methylation platforms, including bisulfite pyrosequencing and Illumina EPIC arrays; analytical pipelines; and clinical food allergy phenotyping. Most candidate-gene and EWAS studies have included relatively small and heterogeneous cohorts, often fewer than 100 participants, and only a limited number of methylation loci have shown partial replication across independent populations. Reproducible changes have most consistently involved *FOXP3*, IL-4, IL-5, and other Th2-regulatory pathways, but cross-study concordance remains modest. Thus, DNA methylation signatures remain promising but exploratory biomarkers requiring further validation.

#### 6.1.2. MicroRNAs Associated with the Allergic Response

MicroRNAs (miRNAs) are short, endogenous, single-stranded non-coding RNA molecules, approximately 18–22 nucleotides in length, that regulate gene expression post-transcriptionally by binding to complementary sequences in the 3′ untranslated region of target messenger RNAs, leading to translational repression or mRNA degradation. Because miRNAs regulate a large proportion of protein-coding genes and remain relatively stable in biological fluids, they are attractive candidates as minimally invasive biomarkers and important modulators of immune cell function.

In allergic disease, miR-21, miR-146a, and miR-155 are among the most extensively studied miRNAs and are consistently dysregulated across several atopic conditions. miR-21 promotes Th2 polarization and dendritic cell differentiation from monocytes, and its upregulation has been documented in murine models of cow’s milk allergy and ovalbumin sensitization [106,107]. miR-146a appears to exert predominantly immunomodulatory effects by limiting follicular T helper cell expansion, upregulating *FOXP3*, and enhancing Treg activity through suppression of the HIPK3/STAT3 axis. This mechanism has been demonstrated in an allergic conjunctivitis model, in which miR-146a targeting of HIPK3 reduced phosphorylated STAT3 and increased *FOXP3* expression in transforming growth factor-β (TGF-β)-induced thymocytes, suggesting a potential role as a molecular brake on type 2 inflammation [108]. miR-155 has a more complex and context-dependent function. It promotes dendritic cell maturation, B-cell-to-plasma-cell conversion, IgE production, and follicular T helper cell differentiation, but may also restrain Th2 cytokine secretion at the T-cell level. This dual activity complicates its interpretation as either a purely pro-allergic or tolerogenic signal.

A systematic review by Rana et al., including both in vivo models and clinical data, identified miR-146a, miR-155, and miR-30a-5p as the miRNAs most consistently dysregulated across food allergy studies [106]. However, the direction of change and the specific molecular targets varied across allergen types, including peanut, cow’s milk, and egg allergy. These findings indicate that no universal food allergy miRNA signature has yet been identified and that allergen-specific and phenotype-specific profiling may be necessary. Other candidates, such as miR-143-3p, have been investigated in peanut allergy and non-celiac wheat sensitivity, while early-life miRNA patterns, including elevated miR-21 in neonatal blood, have been associated with antenatal IgE production and later allergic disease, suggesting potential predictive value during prenatal or early postnatal immune development.

The therapeutic relevance of miRNAs has also been explored in dietary and immunomodulatory interventions. In infants with cow’s milk allergy receiving extensively hydrolyzed casein formula supplemented with *Lactobacillus rhamnosus* GG, differential modulation of miR-155, miR-146a, miR-128, and miR-193a was observed, together with changes in *FOXP3* methylation [109,110]. This coordinated epigenetic response suggests that miRNA regulation and DNA methylation may be mechanistically linked in the immunomodulatory effects of early nutritional interventions. In allergen-specific immunotherapy, miRNA profile changes associated with tolerance induction have also been reported, including upregulation of miR-146a together with increased IL-10 and TGF-β production, a pattern consistent with Treg expansion and broadly aligned with methylation changes observed during OIT [107,111].

Despite these promising findings, the clinical applicability of miRNAs as biomarkers in food allergy remains incompletely defined. Most studies do not report formal diagnostic performance metrics, such as sensitivity, specificity, or area under the receiver operating characteristic curve, limiting assessment of their discriminatory value. Additional challenges include pre-analytical variability related to sample type, including serum, plasma, and whole blood; hemolysis; sample processing and storage; and the absence of universally accepted normalization strategies. Furthermore, although circulating miRNAs are attractive because of their non-invasive accessibility, their relationship with tissue-specific immune events in the gut, skin, or lymphoid tissues remains uncertain. Distinguishing exosomal from cell-free circulating miRNA fractions adds further complexity, as these compartments may differ in biological origin and biomarker relevance.

#### 6.1.3. Evidence Status: Predominantly Exploratory

Despite strong biological plausibility and increasing mechanistic insight, the evidence supporting DNA methylation signatures and miRNA panels as clinically actionable biomarkers in food allergy remains predominantly exploratory. No epigenetic or miRNA-based biomarker has yet achieved the analytical validation, reproducibility, or regulatory endorsement required for routine use in diagnosis, prognosis, risk stratification, or therapeutic monitoring.

Several limitations explain this gap between discovery and clinical application. First, most studies include small and clinically heterogeneous cohorts, often with variable diagnostic criteria for food allergy. Second, analytical platforms differ substantially across studies, including pyrosequencing, methylation arrays, bisulfite sequencing, quantitative reverse transcription PCR, small RNA sequencing, and digital PCR, limiting cross-platform comparability. Third, many studies rely on surrogate tissues such as whole blood, which may not accurately reflect immune events occurring in the intestinal mucosa, skin, or allergen-specific T-cell subsets. Fourth, longitudinal pediatric data remain limited, despite the fact that epigenetic marks evolve dynamically with age, microbiome development, diet, and environmental exposures. Finally, most candidate loci and miRNA signatures have not been independently replicated.

Nevertheless, the field is advancing. The sensitivity of *FOXP3* methylation to OIT-induced tolerance and its reversibility during resensitization provide proof-of-concept that epigenetic biomarkers may be useful for monitoring therapeutic response [103,104]. Machine learning approaches applied to methylation array data are beginning to identify reproducible epigenetic signatures beyond single-locus analyses [105]. In addition, the convergence of miRNA and DNA methylation changes in dietary intervention studies suggests that multi-layered epigenomic panels may ultimately be more informative than single biomarkers [102,103]. Future studies should include adequately powered, longitudinal, multicenter pediatric cohorts, standardized omics platforms, rigorous cell-type annotation, and validated clinical phenotyping to translate epigenomic findings into precision medicine tools.

### 6.2. Multi-Omics Profiling

Multi-omics profiling integrates complementary high-throughput technologies to examine biological systems across multiple regulatory layers, including the genome, epigenome, transcriptome, proteome, metabolome, and microbiome. In pediatric food allergy, these approaches are increasingly used to characterize coordinated alterations in gene expression, protein abundance, metabolic pathways, and microbiome-derived signals that underlie allergic sensitization, clinical reactivity, tolerance acquisition, and response to therapy [112,113]. Rather than focusing on isolated biomarkers, multi-omics analyses reconstruct interconnected molecular networks that link immune dysregulation, epithelial barrier dysfunction, metabolic adaptation, and host–microbiome interactions to clinically observable phenotypes.

Transcriptomic studies conducted during oral food challenges have identified gene expression patterns that correlate with clinical reaction thresholds, including pathways related to Fcγ receptor–mediated phagocytosis and Toll-like receptor signaling, supporting the concept that coordinated gene expression programs contribute to inter-individual variability in allergic responsiveness [114]. Metabolomic profiling of plasma and stool samples collected during infancy and childhood has revealed alterations in lipid species, bile acids, steroid-related metabolites, and sphingolipid pathways in children with food allergy or allergic sensitization. These signatures likely reflect both host metabolic programming and microbiota-driven biotransformation, highlighting the importance of immune–metabolic crosstalk in shaping allergic risk [115,116]. Age-stratified analyses further suggest that metabolic pathways differ across developmental stages, with bile acid and polyamine metabolism more prominent in early life and fatty acid and acylcarnitine perturbations more evident later in childhood, emphasizing the dynamic nature of pediatric food allergy phenotypes [101].

Proteomic approaches, although less extensively studied than transcriptomics and metabolomics, provide complementary information by directly measuring protein expression and post-translational regulation. In pediatric immune-mediated conditions, deep proteome profiling has successfully identified immune-relevant protein signatures and candidate biomarkers, supporting the feasibility of applying similar strategies to food allergy to capture functional immune changes not evident at the transcript or metabolite level [117]. Overall, integrating transcriptomic, proteomic, metabolomic, and microbiome data may generate multidimensional biomarker panels that better reflect the biological complexity of pediatric food allergy than any single data type. Such approaches may enable earlier diagnosis, mechanism-based risk stratification, identification of molecular endotypes, and precision-oriented management strategies [113].

### 6.3. Proteomic Profiling

Proteomic profiling is increasingly being explored in pediatric food allergy as a strategy to identify protein expression patterns that reflect immune perturbation, allergic sensitization, and tolerance development. Because proteins represent functional mediators of immune and epithelial responses, proteomics may provide clinically relevant information that is not fully captured by transcriptomic or metabolomic analyses.

Early plasma proteomic studies in infancy have identified inflammation-related and immune-associated proteins associated with later development of allergic phenotypes, suggesting that systemic protein-level changes may reflect early deviations in immune maturation toward atopy [113]. More targeted high-resolution mass spectrometry analyses have detected distinct serum protein–peptide signatures in atopic children sensitized to plant storage proteins. In these cohorts, proteins involved in cell-cycle regulation and transcriptional repression helped discriminate allergic from non-allergic atopic individuals, suggesting potential utility for early identification of sensitization patterns and risk stratification [118].

Proteomic methodologies have also been applied to specific IgE-mediated food allergies, including cow’s milk allergy, through immunoproteomic mapping of allergen-reactive proteins and antibody-binding profiles. These studies have identified immunodominant milk protein targets and patterns of antibody recognition that distinguish allergic from tolerant pediatric patients, providing mechanistic insight into antigen-specific immune responses and informing the development of molecularly guided diagnostic tools [119]. In parallel, proteomics has been used to characterize the molecular composition of food allergens, including allergenic proteins, isoforms, and IgE-binding epitopes. This high-resolution molecular information supports component-resolved diagnostic strategies and may improve risk assessment by capturing structural and sequence heterogeneity across allergen sources that influence clinical reactivity and cross-sensitization [120].

More recently, metaproteomic analyses of the gut microbial ecosystem have begun to clarify how microbial protein expression relates to mucosal immune regulation and the balance between oral tolerance and allergic sensitization. Microbiota-derived enzymes and structural proteins linked to immune-modulatory metabolic pathways may serve as indirect indicators of host–microbe crosstalk relevant to pediatric food allergy [121]. These findings support the concept that proteomic and metaproteomic signatures could contribute to endotype characterization, particularly when integrated with microbiome and metabolomic data.

Despite these advances, most proteomic biomarkers in food allergy remain at an early translational stage. Several candidate protein signatures have been associated with susceptibility, clinical phenotype, persistence, and tolerance acquisition, but external validation is limited. Inflammation-associated proteins involved in epithelial barrier dysfunction, innate immune activation, complement pathways, acute-phase responses, and Th2-skewed inflammation have been reported in children with IgE-mediated food allergy compared with non-allergic controls. Similarly, serum and cellular proteomic profiles may help distinguish persistent from transient cow’s milk allergy phenotypes. However, these findings remain preliminary and have not yet shown sufficient reproducibility for routine clinical use.

The quantitative strength of current proteomic evidence is also limited. Many studies include small cohorts, use heterogeneous analytical platforms, and lack independent replication. Only a minority reports formal performance metrics, such as fold-change magnitude, receiver operating characteristic (ROC) analyses, sensitivity, specificity, or predictive accuracy. Additional challenges include age-dependent variability in circulating protein profiles, difficulty detecting low-abundance immune mediators in small pediatric samples, and variability in sample processing, mass spectrometry workflows, and bioinformatic pipelines. Standardized protocols, adequately powered multicenter cohorts, and integration with transcriptomic and metabolomic layers are needed to validate proteomic biomarkers and support their clinical implementation for early diagnosis, endotype stratification, tolerance prediction, and personalized management [122,123].

### 6.4. Metabolomic Profiling

Metabolomic profiling captures dynamic changes in small-molecule metabolites generated by immune activation, nutritional exposures, host metabolic programming, and microbial biotransformation. Because metabolites represent downstream products of cellular and microbial activity, metabolomics provides a functional snapshot of biological processes relevant to pediatric food allergy, including epithelial barrier integrity, immune effector function, and microbiota–host metabolic signaling [122].

Untargeted plasma metabolomic studies in IgE-mediated pediatric food allergy have consistently identified perturbations in lipid-associated pathways, including reduced sphingolipids, sphingomyelins, and ceramide species. These lipid classes contribute to membrane architecture and lipid raft organization, both of which are important for antigen presentation and receptor-mediated signaling in immune and epithelial cells. Altered sphingolipid metabolism may therefore reflect impaired epithelial barrier stability and enhanced immune responsiveness during early sensitization [113]. Pathway-based analyses also suggest abnormalities in mitochondrial energy metabolism, including fatty acid β-oxidation and acylcarnitine turnover, indicating that immune cell metabolic reprogramming accompanies allergic inflammation. Such bioenergetic shifts may influence effector T-cell differentiation, cytokine production, and persistence of inflammatory circuits [124].

Age-stratified metabolomic studies indicate that food allergy-associated metabolic perturbations vary across developmental stages. In early childhood, alterations in bile acid composition and polyamine metabolism appear prominent, both of which may influence epithelial turnover and regulatory T-cell differentiation. In older children, dysregulation of long-chain fatty acids and acylcarnitines is more frequently observed, consistent with metabolic adaptation during chronic immune activation. These findings highlight the importance of interpreting metabolomic biomarkers within age-specific biological contexts [100].

Longitudinal birth-cohort analyses have identified early-life endocrine–metabolic patterns associated with later food allergy risk, including inverse associations between androgenic or pregnenolone-derived steroid metabolites and allergic outcomes. Specific phosphoinositol-containing lipids and complex phospholipid species measured during early childhood have also shown predictive value for allergic trajectories, suggesting that membrane lipid remodeling and phosphoinositide signaling may influence immune activation thresholds relevant to food allergy development [116]. Integrated gut metabolome–microbiome profiling in infants with IgE-mediated cow’s milk allergy has shown coordinated depletion of bile acids, short-chain fatty acids, and tryptophan-derived indole metabolites before overt allergic inflammation. These findings implicate impaired microbial metabolic support of regulatory immune pathways in the breakdown of oral tolerance [99].

Collectively, metabolomic data support the concept that pediatric food allergy involves coordinated systemic and intestinal immune–metabolic remodeling driven by both host-intrinsic immune activation and environmentally shaped microbial metabolic outputs. However, clinical translation is limited by marked inter-individual variability, dietary confounding, rapid developmental changes in pediatric metabolism, and heterogeneity across IgE-mediated, non-IgE-mediated, and mixed food allergy phenotypes [122]. Additional methodological challenges include variation in biospecimen type and collection timing, analytical platforms, batch effects, and metabolite annotation pipelines. Most candidate metabolomic biomarkers currently lack standardized pre-analytical protocols, validated diagnostic or prognostic thresholds, and prospective multicenter validation. As a result, metabolomic signatures cannot yet be used as standalone clinical biomarkers. Future studies should prioritize harmonized analytical pipelines, longitudinal sampling, external validation, and integration with proteomic and transcriptomic data to define clinically actionable biomarker panels for diagnosis, prognosis, endotype stratification, and precision-oriented management [113].

### 6.5. Transcriptomic Profiling

Transcriptomic approaches enable detailed characterization of gene expression programs and immune cell activation states involved in allergic sensitization, acute clinical reactivity, and responses to immunomodulatory interventions. By integrating bulk RNA sequencing with cell-resolved profiling strategies, transcriptomics can reveal coordinated immune programs that are not apparent from conventional immunophenotyping alone, offering mechanistic insight into the molecular architecture of pediatric food allergy [123].

Dynamic analyses of whole-blood transcriptomes during double-blind, placebo-controlled oral food challenges in children with peanut allergy have shown that allergen exposure induces rapid and reproducible transcriptional changes that are absent after placebo exposure. These acute allergen-induced signatures include co-regulated gene networks enriched for acute-phase responses, pro-inflammatory mediators, and innate immune activation. Notable peanut-induced transcripts include *LTB4R, PADI4, IL1R2, PPP1R3D, KLHL2,* and *ECHDC3*, which have been proposed as key drivers of transcriptional networks during clinical reactions [124]. Network-based analyses of the same challenge datasets showed that gene modules associated with individual reaction thresholds are enriched for Fcγ receptor–mediated phagocytosis and Toll-like receptor signaling pathways. Their interaction with circulating neutrophil abundance suggests coordinated regulation across innate and adaptive immune compartments and supports a role for transcriptional network architecture in determining variability in clinical reactivity [114].

Beyond acute challenge settings, whole-blood transcriptomic profiling in infants at elevated risk of peanut allergy has identified preclinical alterations in innate immune signaling, including dysregulated type I interferon responses, enrichment of neutrophil-associated gene modules, and reduced regulatory T-cell-associated signatures before overt disease onset [125]. These findings suggest that transcriptional biomarkers may help identify at-risk children during early immune development. Longitudinal transcriptomic analyses in pediatric OIT trials for egg allergy have shown progressive remodeling of gene expression during treatment. Successful desensitization is associated with downregulation of pro-inflammatory and Th17-related pathways and relative enhancement of innate immune response signatures, suggesting that immunotherapy redirects inflammatory transcriptional circuits toward less pathogenic states [126]. Systematic syntheses of transcriptomic data across OIT studies further indicate that durable immunomodulation is characterized by suppression of Th2-skewed transcriptional programs and reinforcement of regulatory networks, including features consistent with T-cell anergy and modulation of type I interferon-associated pathways [123].

Independent studies using weighted gene co-expression network analysis in pediatric nut allergy have identified hub transcripts, such as *IFIH1* and *DRAM1*, within modules associated with type I interferon signaling, neutrophil activation, and reduced regulatory T-cell networks, reinforcing the concept that innate immune bias and impaired regulation contribute to food allergy pathogenesis in early life [125]. Epigenetically informed transcriptomic profiling of naïve CD4^+^ T-cell activation in infants with challenge-confirmed egg allergy has also revealed dysregulation of cell-cycle-associated transcriptional programs, including E2F- and MYC-regulated gene sets, together with remodeling of immune receptor loci such as *IL1R* and *IL18RAP*. These abnormalities correlate with impaired lymphoproliferative responses and may reflect defective oral tolerance induction during critical windows of immune development [126,127].

From a translational perspective, transcriptomic biomarkers in pediatric food allergy can be grouped into four main categories. Diagnostic biomarkers include acute allergen-induced signatures detectable during oral food challenges, characterized by rapid induction of inflammatory and innate immune genes such as *LTB4R, PADI4*, and *IL1R2*. Prognostic biomarkers include early-life transcriptional patterns associated with later disease development or reaction severity, such as type I interferon modules, neutrophil-associated gene networks, and attenuated regulatory T-cell signatures. OIT-response biomarkers include dynamic changes associated with successful desensitization, including suppression of Th2/Th17 inflammatory pathways and reinforcement of regulatory or anergy-associated programs. Finally, endotype-associated transcriptomic signatures may reflect more stable molecular architectures, such as interferon-driven, neutrophil-enriched, or regulatory-deficient subphenotypes, with potential value for precision stratification [124,126].

Despite their promise, transcriptomic biomarkers are not yet ready for routine clinical use. Key challenges include tissue specificity of gene expression, temporal variability between acute reaction and steady-state sampling, differences in RNA sequencing and sample-processing protocols, and heterogeneity in analytical pipelines. Larger multicenter validation studies, standardized transcriptomic workflows, and integration with proteomic, metabolomic, epigenetic, and microbiome data are needed to identify robust transcriptional biomarkers capable of informing early diagnosis, endotype definition, treatment selection, and therapeutic monitoring in pediatric food allergy [123].

### 6.6. Future Directions for Multi-Omics Integration

Converging evidence from epigenomic, transcriptomic, proteomic, metabolomic, and microbiome studies indicates that pediatric food allergy arises from coordinated alterations in immune signaling, epithelial barrier function, metabolic programming, gene regulation, and host–microbiome interactions. These perturbations are dynamic, developmentally regulated, and influenced by environmental exposures, collectively defining biologically distinct disease endotypes. The integration of multi-layered omics data offers a promising route toward biomarker panels that move beyond single-analyte diagnostics and better capture the complexity of pediatric food allergy.

Realizing this translational potential will require rigorous clinical phenotyping, standardized sample collection and analytical pipelines, longitudinal cohort designs, external validation, and broader representation of diverse pediatric populations. Computational approaches, including machine learning and network-based modeling, may help identify reproducible biomarker combinations, but these models must be interpretable, clinically feasible, and validated across independent cohorts. With these advances, multi-omics approaches may help transform pediatric food allergy from a condition defined primarily by clinical history and challenge outcomes into a molecularly stratified disease, enabling earlier diagnosis, more accurate risk prediction, improved monitoring of tolerance acquisition, and personalized therapeutic decision-making.

## 7. Clinical Translation and Future Perspectives

The expanding field of biomarker research in pediatric food allergy reflects the need for tools that improve diagnosis, risk stratification, tolerance prediction, and therapeutic monitoring beyond conventional clinical assessment, skin prick testing, serum-specific IgE, and oral food challenge [15,16,17,18,19,20,21,22]. In this review, biomarkers were appraised using a common translational framework that considered their intended clinical use, biological rationale, available performance data, validation status, reproducibility, feasibility, and readiness for clinical implementation [19,20,21,22].

For diagnostic use, the strongest evidence currently supports functional cellular assays, particularly the BAT [23,24,25,26,27,28,29,30,31,32,33,34,35,36,37,38,39,40,41,42]. BAT provides high specificity in selected IgE-mediated food allergies and may reduce unnecessary oral food challenges in patients with equivocal first-line test results [41,42,43,56,57,61]. Its additional value lies in its capacity to reflect effector cell responsiveness, which may support severity assessment, threshold estimation, and monitoring during oral immunotherapy or biologic treatment [56,67,68,69,70,71,72,73]. However, implementation is constrained by the need for fresh blood, flow cytometry infrastructure, trained personnel, harmonized protocols, standardized allergen preparations, validated food-specific cut-offs, cost-effectiveness data, reimbursement pathways, and regulatory qualification [41,45,46,47,48,49,50,51,52,53,54,55,56,57].

By contrast, epithelial barrier and intestinal permeability biomarkers, including zonulin, intestinal fatty acid-binding protein, diamine oxidase, tight junction proteins, and fecal inflammatory mediators, remain mainly exploratory [74,75,76,77,78,79,80,85]. Their most plausible role is not routine diagnosis, but endotyping of gastrointestinal, non-IgE-mediated, and mixed food allergy phenotypes [19,21,22,75]. Translation is limited by assay variability, uncertain specificity, lack of validated thresholds, small pediatric cohorts, and insufficient longitudinal validation [21,22,75,76,77,78,79,80]. Zonulin illustrates these challenges particularly well: despite being widely studied, concerns regarding the analytical specificity of commercial assays currently restrict its interpretation to a research setting rather than a validated diagnostic application [75,76,77,85].

Genetic and epithelial integrity biomarkers, especially filaggrin loss-of-function variants, are best classified as risk-stratification markers [83,89,90,91,92]. They provide mechanistic insight into barrier dysfunction, atopic dermatitis, transcutaneous sensitization, and subsequent food allergy development [81,82,83,89,90,91,92]. However, because they are static susceptibility markers, they are not suitable for monitoring disease activity, tolerance acquisition, or treatment response unless integrated with dynamic immune, epithelial, microbiota-derived, or metabolomic markers [21,113].

Microbiota-derived and metabolomic biomarkers are promising for tolerance prediction, disease persistence, and endotype identification because they capture host–environment interactions, immune maturation, epithelial barrier function, and oral tolerance mechanisms [93,94,95,96,97]. Reduced microbial diversity, depletion of butyrate-producing taxa, altered short-chain fatty acid profiles, and changes in bile acid or tryptophan metabolism have been associated with food allergy development or persistence [95,96,97,98,99,100]. However, their reproducibility is limited by age, diet, breastfeeding, antibiotic exposure, geography, sample handling, sequencing platforms, metabolomic methods, and bioinformatic pipelines [93,94,95,96,97,98,99,100,113,121]. At present, these biomarkers should be considered investigational rather than clinically actionable [99,100,113,123].

Epigenetic, transcriptomic, proteomic, metabolomic, and integrated multi-omics approaches may offer the greatest long-term potential for precision medicine, but they currently have the lowest clinical readiness [101,102,103,104,105,106,107,108,109,110,111,112,113,114,115,116,117,118,119,120,121,122,123,124,125,126,127]. FOXP3 methylation, selected microRNA profiles, transcriptomic signatures during oral food challenge or immunotherapy, and proteomic or metabolomic patterns may provide insight into immune regulation, clinical reactivity, desensitization, sustained unresponsiveness, and relapse risk [103,104,105,106,111,113,114,115,116,117,118,119,120,121,122,123,124,125,126,127]. However, most studies remain descriptive, include small cohorts, lack standardized platforms, and rarely provide comparable sensitivity, specificity, area under the curve, predictive values, or external validation [101,106,113,121,127]. Therefore, the depth of discussion across biomarker categories necessarily differs: BAT receives greater quantitative emphasis because it has more mature clinical evidence, whereas omics-based markers are discussed mainly as exploratory tools with mechanistic and future translational relevance [23,24,25,26,27,28,29,30,31,32,33,34,35,36,37,38,39,40,41,42,43,56,57,101,102,103,104,105,106,107,108,109,110,111,112,113,114,115,116,117,118,119,120,121,122,123,124,125,126,127].

Across all biomarker classes, clearer distinction is needed between diagnostic, prognostic, predictive, and monitoring applications [19,20,21,22]. A clinically useful diagnostic biomarker should distinguish allergic from tolerant children and reduce unnecessary oral food challenges [15,16,17,18,41,42,56,57,60]. A severity or threshold biomarker should identify patients at risk of severe reactions or low eliciting doses [56,67,68,69,70]. A tolerance-prediction biomarker should help estimate persistence or natural resolution [95,96,97,98,99,100,103,104,113,122]. A treatment-monitoring biomarker should track desensitization, sustained unresponsiveness, relapse risk, or response to oral immunotherapy or biologics [71,72,73,103,104,111,123,126]. Future studies should report evidence separately for each intended use rather than assigning broad clinical value to a biomarker category as a whole [19,20,21,22,113,123].

The major barriers to clinical translation include heterogeneity in patient selection, age ranges, allergens studied, diagnostic confirmation methods, phenotype definitions, sample timing, laboratory protocols, and statistical reporting [19,20,21,22,93,94,95,96,97,98,99,100,113,123]. Future research should prioritize multicenter longitudinal pediatric cohorts, standardized oral food challenge protocols where appropriate, harmonized definitions of IgE-mediated, non-IgE-mediated, and mixed phenotypes, and uniform reporting of sensitivity, specificity, positive and negative predictive values, likelihood ratios, and area under the curve [16,17,18,19,41,42,113,123]. Candidate biomarkers should also be tested against existing clinical tools, including history, skin prick testing, serum-specific IgE, component-resolved diagnostics, comorbid atopic disease, and reaction history, to determine whether they improve clinical decision-making [15,16,17,18,19,20,21,22,41,42].

Practical implementation will require more than biological validity [19,20,21,22]. Biomarkers must be reproducible, affordable, accessible, compatible with pediatric sampling constraints, and supported by clinically interpretable reporting systems [19,20,21,22,41,52,53,54,55,56,57]. Flow cytometry-based BAT requires specialized infrastructure and rapid sample processing [35,46,47,48,49,50,51]. Multi-omics platforms require advanced sequencing or mass-spectrometry facilities, bioinformatic expertise, data integration pipelines, and regulatory frameworks for biomarker qualification [101,102,103,104,105,106,107,108,109,110,111,112,113,114,115,116,117,118,119,120,121,122,123,124,125,126,127]. Reimbursement, turnaround time, cost-effectiveness, quality control, and external proficiency testing will strongly influence whether promising biomarkers can move from research settings into routine pediatric allergy care [19,20,21,22,41,113,123].

Overall, BAT is currently the most clinically advanced biomarker in pediatric food allergy, mainly as a second-line diagnostic and risk-stratification tool [23,24,25,26,27,28,29,30,31,32,33,34,35,36,37,56,57,60,67,68,69,70,71,72,73,74]. Epithelial barrier, genetic, microbiota-derived, metabolomic, epigenetic, transcriptomic, proteomic, and integrated multi-omics biomarkers remain promising but largely investigational [74,75,76,77,78,79,80,81,82,83,84,85,86,87,88,89,90,91,92,93,94,95,96,97,98,99,100,101,102,103,104,105,106,107,108,109,110,111,112,113,114,115,116,117,118,119,120,121,122,123,124,125,126,127]. Their future value will depend on standardized methods, application-specific validation, external replication, practical feasibility, and evidence that they improve clinical outcomes beyond currently available diagnostic approaches [19,20,21,22,101,102,103,104,105,106,107,108,109,110,111,112,113,114,115,116,117,118,119,120,121,122,123,124,125,126,127]. A comparative summary of the translational potential, current limitations, and degree of clinical readiness of emerging biomarkers is provided in Table 3.

**Figure 2:**
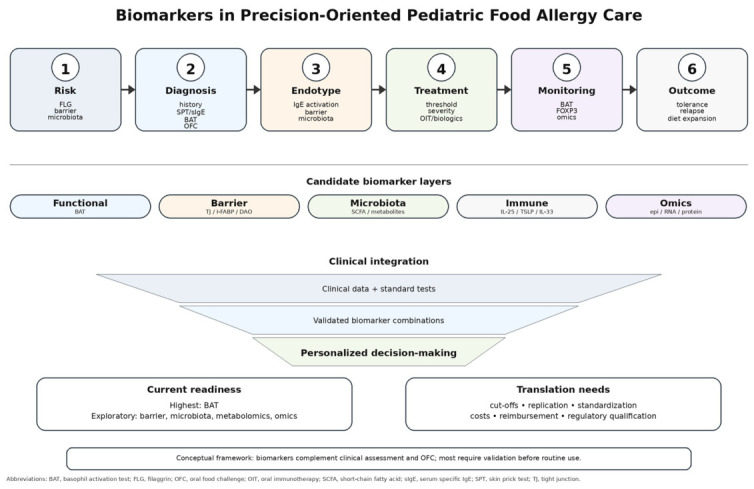
Proposed integration of biomarkers into a precision-medicine framework for pediatric food allergy. Abbreviations: BAT, basophil activation test; CRD, component-resolved diagnostics; epi, epigenetic; FLG, filaggrin; FOXP3, forkhead box P3; I-FABP, intestinal fatty acid-binding protein; IL, interleukin; miRNA, microRNA; OFC, oral food challenge; OIT, oral immunotherapy; RNA, ribonucleic acid; SCFA, short-chain fatty acid; sIgE, serum specific immunoglobulin E; SPT, skin prick test; TJ, tight junction; TSLP, thymic stromal lymphopoietin.

A proposed conceptual framework for integrating biomarker categories into precision-oriented pediatric food allergy care is presented in Figure 2, illustrating their potential roles from early risk prediction and diagnostic refinement to mechanistic stratification, treatment selection, therapeutic monitoring, and outcome assessment.

## 8. Conclusions

Food allergy is a heterogeneous pediatric disease shaped by immune dysregulation, epithelial barrier impairment, genetic susceptibility, environmental exposures, and microbial determinants. This narrative review highlights the expanding role of biomarkers in improving diagnosis, risk stratification, tolerance prediction, therapeutic monitoring, and precision-oriented care.

Among currently available tools, the BAT has the strongest translational readiness. Its high specificity, ability to distinguish sensitization from clinically relevant allergy, and potential value for assessing reaction severity, eliciting thresholds, and treatment response make it useful in selected patients with equivocal first-line test results. However, broader implementation requires standardized protocols, external quality assurance, improved accessibility, and validated food-specific cut-off values.

Other biomarker categories remain less clinically mature. Epithelial barrier and intestinal permeability markers, including zonulin-related pathways, tight junction proteins, epithelial injury markers, FLG variants, and epithelial-derived cytokines, provide important mechanistic insight and may be particularly relevant for gastrointestinal, non-IgE-mediated, and mixed phenotypes. Microbiota-derived, metabolomic, transcriptomic, proteomic, and epigenetic biomarkers may support early-risk identification, tolerance prediction, endotyping, and treatment monitoring. Nevertheless, most remain investigational because of assay variability, heterogeneous study designs, limited pediatric validation, lack of standardized thresholds, and insufficient external replication.

Future progress will require a shift from isolated candidate biomarkers toward integrated, clinically interpretable biomarker panels combining clinical data with functional, barrier-related, microbiota-derived, and omics-based signatures. Large multicenter longitudinal studies, harmonized phenotyping, standardized analytical pipelines, age-specific validation, cost-effectiveness assessment, and demonstration of real-world clinical benefit will be essential. With these advances, validated biomarkers may help move pediatric food allergy care beyond a binary allergic-versus-tolerant model toward more individualized diagnosis, risk assessment, therapeutic selection, and monitoring.

## Figures and Tables

**Table 1:** Overview of emerging biomarkers in pediatric food allergy: biological rationale and potential clinical applications.

Biomarker Category	Main Biomarkers	Biological Rationale	Potential Clinical Utility	Current Validation Status
Functional cellular biomarkers	BAT, CD63, CD203c, CD-sens, EC50	Measures allergen-induced basophil activation and effector cell responsiveness	Diagnosis, reaction threshold prediction, severity stratification, OIT monitoring	Clinically supported; second-line use in selected settings [15,16,17,18,20,21,22]
Intestinal permeability biomarkers	Zonulin, claudins, occludin, ZO-1	Reflect epithelial tight junction dysfunction and increased antigen passage	Disease endotyping, non-IgE phenotype characterization	Limited pediatric validation; no standardized cut-offs [19,20,21,22]
Enterocyte damage biomarkers	I-FABP, DAO	Reflect epithelial injury and mucosal damage	Barrier dysfunction assessment	Limited pediatric validation; no standardized cut-offs [19,20,21,22]
Microbial translocation biomarkers	LBP, soluble CD14, endotoxin-related markers	Indirect markers of increased intestinal permeability/systemic microbial exposure	Experimental mechanistic assessment	Preclinical or early exploratory evidence only [19,20,21,22]
Fecal inflammatory biomarkers	Calprotectin, sIgA, ECP, EDN	Reflect mucosal inflammation and eosinophilic activity	GI food allergy phenotyping	Limited evidence [7,8,9,10,11,19,20,21,22]
Epithelial integrity/genetic biomarkers	*Filaggrin (FLG)* mutations	Barrier dysfunction and transcutaneous sensitization susceptibility	Risk stratification, prediction of persistent/severe disease	Strong mechanistic evidence; not dynamic biomarker [5,6,7,8,20,21,22]
Microbiota/metabolomic biomarkers	SCFAs (especially butyrate), *Clostridiales, Bifidobacterium*	Oral tolerance regulation, immune modulation	Disease prediction, tolerance acquisition	Growing evidence; external validation still limited [20,21,22]

Abbreviations: BAT, basophil activation test; CD, cluster of differentiation; CD-sens, basophil allergen threshold sensitivity; EC50, half-maximal effective concentration; OIT, oral immunotherapy; EAACI, European Academy of Allergy and Clinical Immunology; ZO-1, zonula occludens-1; IgE, immunoglobulin E; I-FABP, intestinal fatty acid-binding protein; DAO, diamine oxidase; LBP, lipopolysaccharide-binding protein; sCD14, soluble cluster of differentiation 14; sIgA, secretory immunoglobulin A; ECP, eosinophilic cationic protein; EDN, eosinophil-derived neurotoxin; GI, gastrointestinal; *FLG, filaggrin*; SCFAs, short-chain fatty acids.

**Table 2:** Diagnostic and prognostic performance of the Basophil Activation Test (BAT) in pediatric food allergy.

Parameter	Findings Reported in Literature	Clinical Implication
Activation markers	CD63, CD203c	Core BAT readouts [23,24,43]
Sample requirement	<0.1 mL fresh blood; ideally within 4 h	Pediatric feasibility [51,52,53,54,55]
Sensitivity	Peanut 86%; Sesame 89%	Good diagnostic sensitivity [41,42]
Specificity	Peanut 90%; Sesame 93%; up to 100% in selected cohorts	High specificity vs. SPT/sIgE [42,43,56,57]
Diagnostic accuracy	96–100% in selected studies	Reduces diagnostic uncertainty [56,57]
Reduction in OFCs	5–15% fewer OFCs	Less invasive work-up [41,42,56,57]
Reduction in positive OFCs	33–75%	Better patient selection [41,42,56,57]
Severity prediction	CD63 correlates with reaction severity	Risk stratification [56]
Threshold prediction	CD-sens, EC50 correlate with eliciting dose	Threshold estimation [56]
Monitoring utility	OIT and omalizumab response monitoring	Therapeutic follow-up [23,24]
Main limitations	Need for fresh blood, flow cytometry, trained personnel, non-responder basophils (~10%)	Limited scalability [41,52,53,54,55,56,57]

Abbreviations: BAT, basophil activation test; CD, cluster of differentiation; CD-sens, basophil allergen threshold sensitivity; EC50, half-maximal effective concentration; OIT, oral immunotherapy; OFC, oral food challenge; sIgE, serum specific immunoglobulin E; SPT, skin prick test.

**Table 3:** Translational readiness of emerging biomarkers in pediatric food allergy.

Biomarker	Main Utility	Strengths	Limitations	Readiness for Clinical Practice *
BAT	Diagnosis, severity, threshold, monitoring	High specificity, OFC reduction, functional assay	Cost, standardization, fresh blood	High (second-line) [23,24,25,26,27,28,29,30,31,32,33,34,35,36,37,38,39,40,41,42,43,44,45,46,56,57,60,65,66,67,68,69,70,71,72,73]
Zonulin	Barrier dysfunction endotyping	Most studied permeability marker	Poor assay specificity, no cut-offs	Low–moderate [21,22,74,75,76,85,86,87]
*FLG* mutations	Risk prediction	Strong genetic association with food allergy/AD	Static susceptibility marker only	Moderate [21,81,82,83,89,90,91,92,93]
SCFAs/butyrate	Tolerance prediction	Mechanistic relevance, microbiota link	High inter-study variability	Low [93,94,95,96,97,98,99,100,122]
Gut microbiota signatures	Endotyping, tolerance prediction	Multi-omics potential	Poor reproducibility, no standardization	Low [93,94,95,96,97,98,99,100,111,113]
IL-25/TSLP/IL-33	Mechanistic endotyping	Strong biologic rationale	Experimental only	Experimental [21,22,74,75,83,84,123]

Abbreviations: AD, atopic dermatitis; BAT, basophil activation test; *FLG, filaggrin*; IL, interleukin; OFC, oral food challenge; SCFAs, short-chain fatty acids; TSLP, thymic stromal lymphopoietin. * Readiness levels are intended as descriptive indicators of current translational maturity rather than formal regulatory grades. “High” indicates biomarkers with reproducible clinical evidence, reported diagnostic-performance metrics, partial integration into guideline-based diagnostic pathways, and potential use in selected clinical settings, although further standardization may still be required. “Moderate” indicates biomarkers with consistent biological plausibility and replicated associations in pediatric or atopic populations, but limited dynamic clinical utility, incomplete validation for decision-making, or no established role in routine diagnostic pathways. “Low–moderate” indicates biomarkers with promising clinical associations but important analytical, methodological, or interpretative limitations, including lack of validated cut-offs, limited external replication, or uncertain assay specificity. “Low” indicates biomarkers supported by emerging or exploratory human data, but without standardized methods, validated thresholds, reproducible performance metrics, or demonstrated clinical utility. “Experimental” indicates biomarkers supported mainly by mechanistic, preclinical, or early exploratory evidence and not currently suitable for routine clinical use.

## Data Availability

No new data were created or analyzed in this study. Data sharing is not applicable to this article.
